# Assessing multiple-choice question quality in internal medicine: a comparative analysis of three large language models against expert consensus

**DOI:** 10.3389/fmed.2026.1866674

**Published:** 2026-07-09

**Authors:** Mevlüt Okan Aydin, Belkıs Nihan Coşkun, İbrahim Hamal

**Affiliations:** 1 Department of Medical Education, Faculty of Medicine, Bursa Uludağ University, Bursa, Türkiye; 2 Division of Rheumatology, Department of Internal Medicine, Bursa Uludağ University Faculty of Medicine, Bursa, Türkiye; 3 Faculty of Medicine, Bursa Uludağ University, Bursa, Türkiye

**Keywords:** assessment quality, bloom’s taxonomy, large language models, medical education, multiple-choice questions

## Abstract

**Background:**

Large language models (LLMs) are increasingly explored for their potential to support quality assurance in medical education assessment. However, limited evidence exists on the alignment between LLM evaluations and expert judgment across multiple dimensions of multiple-choice question (MCQ) quality.

**Methods:**

This comparative methodological study evaluated 85 MCQs from an internal medicine clerkship examination. Three LLMs (Claude Sonnet 4, Gemini 2.5 Flash, and Llama 3.3 70B Instruct Turbo) and three medical education experts independently assessed each question for cognitive level (Revised Bloom’s Taxonomy), alignment with the intended learning outcome (5-point Likert scale), and presence of technical flaws based on NBME guidelines. Agreement was calculated using Fleiss’ kappa for cognitive level classification and Cohen’s kappa for binary technical flaw criteria, with intraclass correlation coefficients (ICC) for Likert-scale alignment ratings.

**Results:**

For cognitive level classification, Gemini (*κ* = 0.424, *p* < 0.001) and Claude (*κ* = 0.415, *p* < 0.001) showed moderate agreement with experts; Llama demonstrated lower agreement (κ = 0.266, *p* < 0.001). Alignment with learning outcomes yielded weak-to-moderate agreement for all models (ICC 0.121–0.380). For technical adequacy, Claude and Gemini achieved almost perfect agreement on detecting negatively worded stems (*κ* = 0.897, *p* < 0.001) and substantial agreement on inconsistent numerical data (κ = 0.661, *p* < 0.001) but showed poor agreement on more subjective flaws. Llama performed poorly across most technical criteria.

**Conclusion:**

Claude and Gemini demonstrate moderate to strong agreement with experts for cognitive level classification and detection of objective technical flaws, suggesting their potential as adjunctive tools in MCQ review. However, weak agreement on learning outcome alignment and variability across models indicates that LLMs cannot yet replace expert judgment. A hybrid approach combining LLM-assisted screening with human expertise may optimize item quality assurance in medical education. These findings derive from a single institution and discipline with a limited item set (*n* = 85) and require multi-center validation before broader generalization.

## Introduction

The quality of assessment instruments is a cornerstone of effective medical education. Multiple-choice questions (MCQs) remain the most widely used format for evaluating medical knowledge at undergraduate, postgraduate, and licensing examination levels, owing to their psychometric robustness, objectivity, and capacity to sample broad content within a limited examination time (1). Well-constructed MCQs can assess a broad range of cognitive processes, from factual recall to complex clinical reasoning, particularly when aligned with appropriate learning objectives and the cognitive levels of Bloom’s taxonomy (2). However, the validity of assessment outcomes depends critically on the quality of individual items. Poorly constructed MCQs introduce construct-irrelevant variance (CIV), a systematic distortion of test scores by factors unrelated to the construct being measured, which may misrepresent examinee competence and threaten the psychometric integrity of high-stakes examinations (3, 4). Recognizing this risk, the National Board of Medical Examiners (NBME) has published detailed item-writing guidelines to minimize technical flaws and ensure that questions measure intended learning outcomes rather than test-taking skills (5). Despite widespread awareness of these guidelines, technical flaws continue to appear in faculty-authored examination items, partly because item-writing training is inconsistent and its effects tend to decay without regular reinforcement (6).

Item development at large-scale licensure and certification organizations differs markedly from that within individual academic institutions. National testing bodies such as the National Board of Medical Examiners (NBME) and the National Board of Osteopathic Medical Examiners (NBOME) construct items through a resource-intensive pipeline of repeated expert review and empirical pretesting, administering candidate items as unscored questions and analyzing them statistically across testing cycles before operational use, so that weak or flawed items are revised or discarded before they can affect scores (5). Individual medical schools rarely have comparable psychometric infrastructure or timelines; their items are usually written by the same faculty who teach the content and are reviewed, if at all, through heterogeneous and often informal procedures (7), leaving locally developed examinations more vulnerable to construct-irrelevant variance and to underrepresentation of higher-order cognitive skills (3, 8). This asymmetry in review capacity is precisely what makes scalable, low-cost approaches to item quality assurance especially valuable for academic institutions, which must safeguard assessment quality without the extensive review apparatus available to national testing bodies.

In response to this difficulty, the emergence of large language models (LLMs) has attracted considerable interest across the health professions education community. LLMs have demonstrated broad capabilities in medical knowledge retrieval, clinical reasoning support, text summarization, and examination question generation (9, 10). Several studies have explored LLMs’ capacity to generate MCQs or evaluate cognitive alignment in educational content (11–13).

Most published studies in this area have evaluated a single LLM or have employed non-standardized evaluation criteria, limiting generalizability (12, 14). Moreover, the comparative performance of different LLM architectures across multiple quality dimensions, cognitive level, learning-outcome alignment, and technical adequacy has not been rigorously examined using validated rubrics grounded in established item-writing guidelines. Closing this research gap is essential for determining whether LLMs can be reliably integrated into item-review workflows in medical education.

This study, therefore, aims to compare the performance of three state-of-the-art LLMs (Claude Sonnet 4, Gemini 2.5 Flash, and Llama 3.3 70B Instruct Turbo) with that of three human experts in evaluating the quality of MCQs from an internal medicine clerkship examination. Using the NBME technical flaw criteria and the Revised Bloom’s Taxonomy as reference frameworks, we assess inter-rater agreement between each LLM and an expert-derived reference standard across three dimensions: cognitive level classification, alignment with learning outcomes, and presence of specific technical flaws. By characterizing the strengths and limitations of each model, we aim to provide evidence-based guidance on the potential role of LLMs in the quality assurance of medical assessment items.

## Materials and methods

### Study design

This study is a comparative methodological investigation designed to evaluate the quality of 85 multiple-choice questions derived from a written examination administered during the internal medicine clerkship. It examines the performance of large language models (LLMs) in evaluating examination questions, comparing their evaluations with those provided by expert academicians in medical education.

### Study groups

Two primary evaluator groups were established. The experimental group consisted of three distinct large language models: Claude Sonnet 4, Gemini 2.5 Flash, and Llama 3.3 70B Instruct Turbo. The three LLMs were selected because they represented state-of-the-art, publicly accessible models developed by different organizations and widely used across educational and research settings. Using models from different providers allowed evaluation across distinct architectures and training approaches rather than focusing on a single vendor. In addition, all three models supported API-based access, enabling standardized JSON-formatted outputs and ensuring consistency in the evaluation process. The control group was comprised of three faculty members holding doctoral degrees in medical education.

### Evaluated material

The sample pool for this study consisted of 100 multiple-choice questions from the end-of-clerkship written examination for the first rotation group in the internal medicine clerkship during the 2025–2026 academic year. Of these, two questions were excluded because they required interpretation of radiological images; one was excluded for including visual content, and 12 were excluded because they did not correspond to any learning outcome defined in the National Core Education Program (UÇEP). The remaining 85 questions constituted the study sample. For each question, a structured dataset was created, including the question number, the stem, the targeted UÇEP learning outcome, the correct answer choice, and the text of all options A–E. These items had been developed locally by the faculty responsible for the clerkship as part of routine assessment practice and had not undergone formal external validation prior to this study. Only item content was analyzed; no student responses, scores, or other identifying data were used, and all items were de-identified prior to evaluation.

To assess the technical quality of the questions, the “technical flaws” section from the American National Board of Medical Examiners (NBME) guidelines for writing multiple-choice questions was translated into Turkish and utilized as a reference document in the evaluation process. The technical flaws identified in this guideline and used as the basis for analysis were: long and complex options, unnecessarily complex stems, inconsistent numerical data, vague terms, use of “none of the above,” structurally inconsistent options, negatively worded stems, all-inclusive options, absolute terms, grammatical cues, conspicuous correct answers, word repeats, and common words pointing to the correct answer.

### Evaluation dimensions

Both evaluator groups (three experts and three LLMs) independently and blindly assessed the 85 questions across the following three dimensions:

*Cognitive level*: The cognitive level of each question was classified according to the six-tier Revised Bloom’s Taxonomy. These tiers are: Remember, Understand, Apply, Analyze, Evaluate, and Create.
*Alignment with learning outcome*: The adequacy of each question in measuring its intended learning outcome was rated on a 5-point Likert scale (1: Inadequate, 5: Excellent).
*Technical adequacy*: Questions were examined for technical flaws as defined in the NBME guidelines, with each flaw category marked as present or absent (Yes/No).

An evaluation rubric with identical content was used for both experts and LLMs. The full text of this rubric is provided in Supplementary material 1.

### Configuration of large language models

API calls were executed via Google Apps Script within Google Sheets. The models were operated using the default temperature value (0.7). The three LLMs employed in this study were run using the Chain-of-Thought prompting method, in which the models were instructed to reason step by step before reaching their final evaluation.

Each API call was made using a prompt structure consisting of two components. The first part of the prompt contained the Turkish translation of the NBME technical flaws guideline in plain text format. The second part transmitted the question text, correct answer, options, and targeted learning outcome to the model, structured in JSON format. The model was also instructed to generate its response in accordance with a predefined JSON schema. The complete prompt, comprising the instruction block, the per-item data input, and the required JSON output schema, is provided in Supplementary material 2.

Each API call was conducted independently without retaining prior conversation history, thereby preventing the model’s responses from influencing one another. To assess consistency, the same prompt was repeated three times per question, and the most frequent result among these three responses was adopted as the final decision for that question. In cases where no majority was reached among the three repetitions, the lower cognitive level was chosen for Bloom’s taxonomy, and the lower score was selected for the Likert scale.

### Expert evaluation process and expert-derived reference standard determination

The three expert evaluators assessed the questions independently. In cases where all three experts agreed, that value was directly accepted as the expert-derived reference standard. In instances of disagreement, the majority vote (the consensus of two or more of the three experts) was established as the expert-derived reference standard. When no majority was achieved, the lower cognitive level was selected for Bloom’s taxonomy, and the lower score was adopted for the Likert scale. Selecting the lower category in tied cases provided a conservative tie-breaking rule that avoided overstating the cognitive demand or learning-outcome alignment of an item. The expert consensus obtained through this method served as the reference criterion for the statistical analyses. This approach was chosen to preserve the independence of expert judgments and to establish a reproducible expert-derived reference standard. A formal consensus procedure, such as the Delphi method, was not used because the study aimed to compare LLM outputs with independent expert evaluations rather than to obtain discussion-based consensus ratings.

### Statistical analysis

Descriptive statistics were reported as median (minimum–maximum) for quantitative variables, and as frequency for categorical variables. For quantitative data, intraclass correlation coefficients (ICC) with 95% confidence intervals (CI) were calculated to assess inter-rater reliability (15). Since the expert raters were assumed to represent a larger population rather than being fixed, the ICC (1, 2) model with two-way random effects, absolute agreement, and single measurement was applied.

For categorical data, inter-rater agreement was evaluated using Kappa statistics. Cohen’s Kappa (*κ*) was used for binary or nominal variables with two categories (16), while Fleiss’ Kappa (κF) was employed for multi-level categorical variables such as cognitive levels in Bloom’s taxonomy, where more than two categories were present (17). Both coefficients were reported with corresponding 95% CI and were interpreted according to the benchmark thresholds proposed by Landis and Koch (18).

Agreement between expert consensus and large language models (Claude, Gemini, Llama) was assessed across three domains:

- Cognitive Level (Bloom’s taxonomy) evaluated using Fleiss’ Kappa.
- Alignment with Learning Outcomes (Likert scale ratings) evaluated using ICC values.
- Technical Appropriateness (categorical indicators such as presence of complex options, inconsistent numerical data, ambiguous terms, etc.) evaluated using Cohen’s Kappa.

For continuous measures (e.g., Likert scores), ICC values were reported. For categorical measures, *κ* coefficients (Cohen or Fleiss, depending on the number of categories) were calculated. In cases where at least one rater provided identical responses across all items, resulting in zero variance, *κ* values could not be computed. These instances are indicated in the tables as “–*.” *p* < 0.05 was considered significant. Statistical analyses were performed with IBM SPSS ver.23.0 (IBM Corp. Released 2015. IBM SPSS Statistics for Windows, Version 23.0. Armonk, NY: IBM Corp.).

## Results

### Descriptive statistics of evaluations

The distribution of evaluations for cognitive level, alignment with learning outcomes, and technical adequacy criteria is presented in Table 1.

**Table 1:** Frequency distribution of evaluations by experts and large language models.

		Claude	Gemini	Llama	Expert
Cognitive level	Analyze	0	0	0	2
	Understand	33	6	0	31
	Evaluate	32	24	29	24
	Remember	7	19	3	28
	Apply	13	36	53	0
Alignment with learning outcome (median, min–max)		3 (2–5)	4 (2–5)	5(3–5)	3(1–4)
Technical adequacy	Response				
Long or complex options	No	81	83	84	80
	Yes	4	2	1	5
Inconsistent numerical data	No	83	83	85	84
	Yes	2	2	0	1
Vague/ambiguous terms	No	82	84	85	82
	Yes	3	1	0	3
‘None of the above’ option	No	85	85	85	85
Structurally inconsistent options	No	81	84	85	77
	Yes	4	1	0	8
Unnecessarily complex stem	No	83	85	85	83
	Yes	2	0	0	2
Negatively worded stem	No	53	53	82	57
	Yes	32	32	3	28
Grammatical cues	No	84	85	83	84
	Yes	1	0	2	1
All-inclusive options	No	85	82	85	71
	Yes	0	3	0	14
Absolute terms	No	79	80	85	81
	Yes	6	5	0	4
Conspicuous correct answer	No	78	71	83	71
	Yes	7	14	2	14
Word repeats	No	81	84	85	85
	Yes	4	1	0	0
Common words pointing to the correct answer	No	84	84	85	85
	Yes	1	1	0	0

Descriptive statistics are presented as median (min–max) and n.

#### Cognitive level

Experts classified the largest proportion of questions as “Understand” (*n* = 31) and “Evaluate” (*n* = 24). Claude most frequently assigned “Understand” (*n* = 33) and “Evaluate” (*n* = 32), while Gemini predominantly classified questions as “Apply” (*n* = 36). Llama assigned “Apply” to the majority of questions (*n* = 53).

#### Alignment with learning outcome

Median scores (min–max) were 3 (1–4) for experts, 3 (2–5) for Claude, 4 (2–5) for Gemini, and 5 (3–5) for Llama.

#### Technical adequacy

For most technical flaw criteria, the frequency of “Yes” (i.e., presence of a flaw) was low across all evaluators. Notable exceptions were “negatively worded stem,” where Claude (*n* = 32), Gemini (*n* = 32), and experts (*n* = 28) identified the flaw in a substantial number of questions, and “structurally inconsistent options,” which experts flagged in 8 questions while LLMs rarely did. For certain criteria (e.g., “‘None of the Above’ Option,” “word repeats,” “common words pointing to the correct answer”), at least one evaluator recorded no instances of the flaw across all 85 questions.

### Agreement between experts and large language models

Inter-rater reliability among the three expert evaluators was examined before establishing the expert-derived consensus reference standard. For Bloom’s taxonomy classification, agreement among experts was low (Fleiss’ *κ* = 0.117, *p* = 0.001). Unanimous agreement was observed in 11 of 85 items, majority agreement in 57 items, and no majority agreement in 17 items.

For learning-outcome alignment ratings, expert agreement was also poor. The single-measure ICC based on a two-way random-effects absolute-agreement model was 0.089 (95% CI: −0.010 to 0.210, *p* = 0.012), and the average-measure ICC was 0.226 (95% CI: −0.031 to 0.440, *p* = 0.012). Unanimous agreement was observed in only 1 item, majority agreement in 35 items, and no majority agreement in 49 items.

For technical flaw detection, expert agreement varied substantially across criteria. The highest agreement was observed for negatively worded stems (Fleiss’ *κ* = 0.751, *p* < 0.001), followed by inconsistent numerical data (*κ* = 0.488, *p* < 0.001), absolute terms (*κ* = 0.283, *p* < 0.001), and long or complex options (*κ* = 0.235, *p* < 0.001). For several low-prevalence or more subjective flaw categories, kappa values were low or negative despite moderate to high percentage agreement, suggesting that the interpretation of kappa was influenced by prevalence imbalance and the subjective nature of these criteria (Supplementary Table 1).

To address the low prevalence of several individual technical flaw categories, a composite “any technical flaw” variable was created. According to the expert-derived consensus reference standard, at least one technical flaw was identified in 50 of 85 items (58.8%). Claude identified at least one flaw in 55 items (64.7%), Gemini in 56 items (65.9%), and Llama in only 7 items (8.2%).

For the composite “any technical flaw” outcome, agreement with the expert-derived consensus reference standard was moderate for Claude (*κ* = 0.528; agreement = 77.6%) and Gemini (*κ* = 0.551; agreement = 78.8%), whereas Llama showed minimal agreement (*κ* = 0.036; agreement = 44.7%). Claude and Gemini demonstrated high sensitivity for detecting items with at least one technical flaw (86.0 and 88.0%, respectively), while Llama showed very low sensitivity (10.0%) despite high specificity (94.3%) (Supplementary Table S2).

The total number of technical flaws identified per item was moderately correlated with the expert-derived flaw count for Claude (Spearman’s *ρ* = 0.547, *p* < 0.001) and Gemini (*ρ* = 0.542, *p* < 0.001), but not for Llama (*ρ* = 0.113, *p* = 0.302). These composite analyses provided a more stable summary of technical adequacy than criterion-specific kappa estimates for rare flaw categories (Supplementary Table S3).

As presented in Table 2, agreement between the experts and each of the three large language models (Claude, Gemini, and Llama) was examined across the dimensions of cognitive level, alignment with learning outcomes, and various technical adequacy parameters using Cohen’s kappa (*κ*) and the intraclass correlation coefficient (ICC). For cognitive level classification, Gemini (*κ* = 0.424, *p* < 0.001) and Claude (*κ* = 0.415, *p* < 0.001) demonstrated the highest agreement with the experts, corresponding to moderate agreement, whereas Llama exhibited lower agreement (*κ* = 0.266, *p* < 0.001). In the assessment of alignment with learning outcomes, all models showed statistically significant but weak-to-moderate agreement with the experts (ICC = 0.380 for Claude, 0.218 for Gemini, and 0.121 for Llama).

**Table 2:** Assessment of agreement between experts and large language models.

	Agreement	Claude- expert		Gemini- expert		Llama- expert	
Cognitive level (Bloom’s taxonomy)	κ(95%CI); *p*	0.415	<0.001	0.424	<0.001	0.266	<0.001
Alignment with learning outcomes	ICC(95%CI); *p*	0.380	<0.001	0.218	<0.001	0.121	0.009
Technical adequacy							
Long or complex options	*κ*(95%CI); *p*	0.179	0.096	0.557	<0.001	−0.020	0.801
Inconsistent numerical data	κ(95%CI); *p*	0.661	<0.001	0.661	<0.001	–	–*
Vague/Ambiguous Terms	κ(95%CI); *p*	0.309	0.004	−0.018	0.847	–	–*
‘None of the above’ option	*κ*(95%CI); *p*	–	–*	–	–*	–	–*
Structurally inconsistent options	κ(95%CI); *p*	−0.067	0.509	−0.021	0.746	–	–*
Unnecessarily complex stem	κ(95%CI); *p*	0.488	<0.001	–	–*	–	–*
Negatively worded stem	κ(95%CI); *p*	0.897	<0.001	0.897	<0.001	0.139	0.012
Grammatical cues	κ(95%CI); *p*	−0.012	0.913	–	–*	−0.016	0.876
All-inclusive options	κ(95%CI); *p*	–	–*	0.188	0.017	–	–*
Absolute terms	κ(95%CI); *p*	0.576	<0.001	0.414	<0.001	–	–*
Conspicuous correct answer	κ(95%CI); *p*	0.091	0.368	0.059	0.584	−0.043	0.525
Word repeats	κ(95%CI); *p*	–	–*	–	–*	–	–*
Common words pointing to the correct answer	κ(95%CI); *p*	–	–*	–	–*	–	–*

*Value could not be calculated because at least one evaluator provided the same response across all items, resulting in zero variance.

A detailed analysis of the technical adequacy criteria revealed that Claude and Gemini achieved almost perfect agreement with the experts on the item “negatively worded stem” (*κ* = 0.897, *p* < 0.001). For the criterion “inconsistent numerical data,” these two models also demonstrated substantial agreement (κ = 0.661, *p* < 0.001). In contrast, the Llama model either showed disagreement (negative or non-significant kappa values) or yielded incalculable estimates due to the absence of variance among evaluators for the majority of technical adequacy criteria. Furthermore, for certain technical flaws, such as “‘None of the Above’ Option,” “word repeats,” and “common words pointing to the correct answer,” kappa coefficients could not be calculated for any of the models, as at least one evaluator (either the experts or one of the AI models) gave the same response across all questions in the dataset (zero variance).

## Discussion

Taken together, the findings of this study align with the broader trajectory of the LLM-in-education literature: models can approximate human judgment on tasks that are structurally well-defined and amenable to pattern recognition, yet fall meaningfully short when evaluation requires curriculum-specific knowledge, contextual reasoning, or subjective pedagogical appraisal. To our knowledge, this study is among the first to position LLMs not as examinees answering MCQs, but as structured evaluators of item quality, a role that is methodologically distinct from most published work in the field and arguably of greater practical relevance to assessment quality assurance.

Regarding cognitive level classification, the moderate agreement achieved by Claude and Gemini is broadly consistent with prior investigations into LLM performance on Bloom’s taxonomy-based tasks. One study examining GPT-4’s errors on psychosomatic medicine examination questions through the lens of the Revised Bloom’s Taxonomy found that the model’s mistakes were concentrated at the higher cognitive levels, particularly those requiring application and analysis, suggesting inherent limitations in accurately navigating the full taxonomic hierarchy (19). Similarly, another study benchmarking ChatGPT-4 against medical students across varied Bloom’s levels reported that the model’s performance advantage over human examinees was largely confined to lower cognitive categories, such as remembering and understanding, and diminished substantially at higher-order levels (20). The systematic overassignment of questions to the Apply category by Llama, observed in the present study, reflects a mid-range cognitive level bias identified in a large-scale analysis of LLM evaluation benchmarks using the Bloom’s framework: models trained on general corpora tend to over-represent middle taxonomic levels while under-representing both the lowest and highest cognitive categories (21). Finally, an evaluation of five LLMs in generating taxonomy-aligned dental MCQs reported that Claude Sonnet 4 demonstrated superior alignment at higher cognitive levels compared to competing models, including ChatGPT-4o and DeepSeek R1, a finding that echoes the comparatively stronger Bloom’s classification performance of Claude observed in the present study (22).

These agreement levels are best interpreted in the context of the substantial variability that characterizes expert human judgment itself, rather than against an infallible standard. Classifying items by cognitive level is a notoriously difficult and subjective task even for trained faculty: in one study, pharmacy faculty assigning examination questions to Revised Bloom’s categories achieved only slight inter-rater agreement (*α* = 0.25) and correctly classified fewer than half of the items (46%), with accuracy improving to over 80% only when the six levels were collapsed into three broader tiers (23). Comparable variability affects the technical dimension: in the absence of targeted faculty development, faculty-authored items frequently contain item-writing flaws and disproportionately sample lower cognitive levels, whereas structured training has been shown to increase the proportion of higher-order items and to reduce item-writing flaws and nonfunctioning distractors (6, 24). Against this backdrop, the disagreements among our three experts, which in a number of items precluded a majority and required application of the predefined decision rule, are therefore consistent with this literature and reflect an inherent limitation of fine-grained Bloom’s classification rather than a deficiency unique to this panel. Indeed, the moderate LLM-expert agreement achieved by Claude and Gemini is of a similar order to the faculty-faculty agreement reported in such work.

The weak-to-moderate agreement observed across all models on the learning outcome alignment dimension is consistent with evidence from studies examining LLM performance on rubric-based grading tasks that require cognitive differentiation. One evaluation of GPT-3.5, GPT-4o, and Gemini on student assignments graded according to Bloom’s cognitive domains in a sports physiology course found that while models performed reasonably well at the Understand level, statistically significant divergences from human rater scores emerged at the Analyze and Evaluate levels, precisely the domains that demand deeper contextual and disciplinary knowledge (14). The present study’s alignment dimension extends this challenge further: rather than grading the quality of a student’s answer, the task requires determining whether a question genuinely operationalizes the curricular objective to which it has been assigned. This involves institutional knowledge of the intended learning outcomes and the pedagogical logic of the assessment design, a form of curricular expertise grounded in the principles of constructive alignment that is not derivable from text processing alone (25). A systematic review of LLM applications in medical education similarly concluded that while models demonstrate surface-level content competency, judgments about content validity and curricular alignment continue to depend on human oversight (10).

The technical flaw analysis yields the most practically actionable findings of the study. The near-perfect agreement between Claude and Gemini and the expert panel on the detection of negatively worded stems, together with the substantial agreement on inconsistent numerical data, suggests that these two models are reliably sensitive to surface-structural properties of MCQ items, properties that can be identified through syntactic pattern recognition without requiring deep content understanding. This profile is consistent with findings from docimological quality analyses of LLM-generated MCQs, which have demonstrated that LLMs using GPT-based services were able to flag structurally defined item-writing guideline violations, including grammar cues and implausible distractors, with reasonable consistency, but performed far less reliably on criteria that required evaluating the plausibility of content in relation to the clinical or scientific domain (26). The present study’s finding that both Claude and Gemini failed to achieve significant agreement with experts on flaws such as conspicuous correct answers or structurally inconsistent options mirrors this pattern: these criteria require the evaluator to assume the perspective of a candidate taking the examination and to assess the inferential transparency of the item, a task that exceeds what surface-level linguistic analysis can accomplish. The theoretical underpinning of this distinction is well-articulated in the LLM-as-evaluator literature, which consistently demonstrates that classification tasks are more tractable for LLMs than generative tasks, but that even classification degrades when the decision boundary is pragmatic rather than syntactic (27, 28).

A useful conceptual lens is to distinguish four functional roles for LLMs in medical assessment: as examinees (answering questions), as item generators (creating new MCQs), as automated graders (scoring student responses), and as quality appraisers (reviewing item construction) (Box 1). As examinees, LLMs have shown impressive performance, frequently matching or surpassing medical students on standardized exams. One study found that four LLMs matched or exceeded average student performance on Clinical Pharmacology and Therapeutics examinations, significantly outperforming students on both knowledge and prescribing skills components of the European Prescribing Exam (27). Critically, the same study also documented that about a quarter of LLM errors on locally authored examinations were attributable not to genuine knowledge gaps but to ambiguous or erroneously keyed MCQ items. When both ChatGPT and Gemini gave the same theoretically incorrect answer, the specificity for identifying a genuinely flawed item reached 92.9%. Extending these findings, a large-scale comparison of GPT-4, GPT-4o and Google Bard across four international licensing examinations reported accuracy rates exceeding 90% on USMLE steps for GPT-4o (29).

BOX 1 **Four functional roles of large language models in medical assessment.**

**Table:** 

Role	Characterisation in medical assessment
Examinee (answering questions)	Answers examination questions; leading models frequently match or surpass medical students on standardized examinations.
Item generator (creating new MCQs)	Produces new multiple-choice questions. Output is often educationally usable but of variable quality and typically requires human post-editing before use.
Automated grader (scoring responses)	Scores student responses, including free-text answers. Performance is markedly variable and domain-sensitive, generally reaching only moderate agreement with human graders.
Quality appraiser (reviewing item construction)	Evaluates existing items for construction quality, cognitive level, learning-outcome alignment, and technical flaws. This is the role examined in the present study; reliability is highest for objective, rule-based criteria and declines for contextual, curriculum-specific judgments.

As item generators, LLMs have demonstrated the capacity to produce educationally acceptable MCQs, although the quality remains variable and typically requires human post-editing. One recent evaluation of Claude 3.5 Sonnet, GPT-4o, and Gemini 1.5 Pro for generating MCQs from clinical vignettes found that a substantial proportion of generated items contained technical flaws such as implausible distractors or inconsistent keying, yet the most capable models reached acceptable quality after light human revision (13). This generative role is distinct from the quality appraisal function examined here, but it shares a common requirement: the need for structured, criterion-based evaluation of item properties.

As automated graders of student free-text responses, LLM performance is markedly more variable and domain-sensitive. One evaluation of five LLMs grading 804 medical short-answer responses across four basic science disciplines found that no single model performed consistently well across all domains, and providing expert rubrics paradoxically degraded performance in several cases (28). A separate large-scale study grading 2,288 student answers from 12 undergraduate medical courses using GPT-4 and Gemini 1.0 Pro confirmed this pattern: both models reached only moderate agreement with human graders, with GPT-4 proposing significantly lower grades than human evaluators (30). That no model reliably replicated expert-level short-answer grading mirrors our finding for learning outcome alignment, a similarly open-ended and contextually demanding task.

The present focus on MCQ item quality appraisal occupies a fourth, underexplored position, requiring LLMs to function as structured evaluators of item construction rather than as knowledge retrievers, item generators, or response graders. Across all four roles, the same capability boundary emerges: LLMs perform most reliably when the evaluation criterion is syntactically or factually defined; reliability degrades as the task shifts toward contextual judgment, partial-credit discrimination, and curriculum-specific content validity. Thus, integrating LLMs into MCQ quality assurance workflows is most defensible as a targeted, criterion-specific application rather than a comprehensive substitute for expert review. Objective, rule-based technical flaws represent a plausible high-value target for LLM-assisted screening, because their detection does not require disciplinary expertise and the cost of undetected flaws, compromised measurement validity, and unfair examination conditions, is substantial (6). Conversely, cognitive level classification and especially learning outcome alignment remain domains where human expertise is indispensable, at least with current-generation models. A structured hybrid workflow, LLM screening as a first pass that flags structurally defined flaws for confirmation, and content-validity judgments escalated to expert review represent a pragmatic model for translating these findings into practice (31).

Beyond prompt-based methods, the performance limitations observed here, particularly for Llama and for the more contextual evaluation dimensions, may be amenable to improvement through machine-learning strategies that adapt models to the specific task. Domain-specific fine-tuning has repeatedly narrowed the gap between general-purpose models and expert performance in medicine; Med-PaLM 2, which combined medical-domain fine-tuning with refined reasoning and grounding strategies, reached accuracies above 85% on medical licensing-style questions and produced long-form answers that physicians frequently rated comparably to those of other physicians (32). A range of practical adaptation techniques, supervised fine-tuning on expert-annotated examples, parameter-efficient fine-tuning of open-weight models such as Llama, and retrieval-augmented generation that grounds model outputs in authoritative item-writing guidelines, has been described specifically for specialized medical applications and could be applied to item-quality appraisal (33). Fine-tuning an open-weight model on a corpus of expert-annotated examination items, in particular, offers a plausible route to improving agreement on the dimensions that proved most difficult here while retaining the cost and data-governance advantages of locally deployable models. Crucially, these approaches are most realistic as components of a human-in-the-loop system rather than as autonomous replacements: even high-performing fine-tuned medical models require expert review of their outputs to ensure validity and safety, reinforcing a hybrid model in which AI-generated screening is confirmed by expert judgment (10, 31, 33).

## Limitations

This study has several limitations. First, it used a single dataset of 85 MCQs from the internal medicine clerkship examination of one medical faculty, which restricts the generalizability of the findings to other disciplines, item formats, and institutional contexts; the small number of items also limited the reliability of statistical analysis for low-prevalence technical flaws. Second, the reference standard was derived from the consensus of three human experts and therefore does not represent absolute truth; notably, inter-rater agreement among the experts was low for Bloom’s taxonomy classification and learning-outcome alignment, so the expert-derived standard should be interpreted as a consensus among independent raters rather than an absolute criterion, particularly for these more subjective dimensions. Third, student-level response data were not available, so classical item statistics such as the difficulty index and corrected point-biserial correlation could not be calculated; these indices would have provided complementary evidence on the empirical functioning of the items and on whether expert- or LLM-identified flaws are associated with reduced discrimination or atypical difficulty. The evaluation rubric was based on a Turkish translation of the NBME item-writing guidelines. While maximum fidelity to the original was maintained, the possibility of minor interpretive differences affecting the results cannot be ruled out entirely. Finally, the models were run with the temperature parameter at its default value (0.7) and were deployed using zero-shot prompting without task-specific fine-tuning for medical education; although triplicate sampling with majority vote mitigated response variability, different sampling, prompting, or tuning strategies might have yielded different outcomes.

## Future directions

Future research should explore several avenues. First, expanding the item pool to include multiple examinations across different disciplines would enhance generalizability and enable more robust statistical comparisons for low-prevalence flaws. Second, combining expert and LLM-based item appraisal with student response data would enable the incorporation of classical item statistics, supporting a more comprehensive psychometric validation of MCQ quality. Investigating the effects of different prompting strategies, temperature settings, and few-shot learning approaches could help optimize LLM performance for item evaluation. Fourth, prospective studies examining whether LLM-based feedback improves item quality over time would provide evidence of practical utility. Cross-cultural validation of translated rubrics or the use of original-language guidelines would help confirm that the findings are not affected by translation. Finally, as LLM capabilities continue to evolve, ongoing evaluation of newer model versions will be necessary to determine whether performance gains translate to improved alignment with expert judgment.

## Conclusion

This study demonstrates that large language models vary considerably in their ability to evaluate the quality of multiple-choice questions. Claude and Gemini show moderate to strong agreement with expert consensus for cognitive level classification and for detecting certain rule-based technical flaws, suggesting their potential as adjunctive tools in item review processes. However, their performance in assessing alignment with learning outcomes remains weak, and Llama consistently underperforms across all dimensions. These findings indicate that while LLMs can support certain aspects of item quality assurance, they cannot currently replace human expertise. A hybrid approach, leveraging LLMs for rapid, objective screening while retaining expert judgment for complex, content-dependent evaluations, may represent the most prudent path forward for integrating artificial intelligence into medical education assessment workflows.

## Data Availability

The raw data supporting the conclusions of this article will be made available by the authors, without undue reservation.
