# Supplementary material for: Assessing multiple-choice question quality in internal medicine: a comparative analysis of three large language models against expert consensus
Source: Front Med (Lausanne). 2026 Jul 9;13:1866674. doi: 10.3389/fmed.2026.1866674 (PMC13391911; doi:10.3389/fmed.2026.1866674)
Supplement: Supplementary file 1 [file Data_Sheet_1.docx]

*Supplementary Material 1*

**Item Evaluation Rubric**

This form was developed to evaluate the educational and technical quality of the multiple-choice questions (MCQs) used in the Internal Medicine Clerkship End-of-Rotation Written Examination, with respect to established item-writing standards.

| **Evaluator Group** | Faculty Member in Medical Education / Large Language Model (LLM) |
| --- | --- |
| **Examination** | Internal Medicine Clerkship — End-of-Rotation Written Examination |
| **Question Stem** |  |
| **Correct Answer** |  |
| **Target Learning Outcome (UÇEP)** |  |
| **Answer Options (A–E)** |  |

# 1. Cognitive Level Classification

Identify the cognitive level that the question requires the student to demonstrate. Select only one category.

| **Category** | **Definition** | **Selection** |
| --- | --- | --- |
| **Remember** | Questions requiring the student to retrieve previously learned information from memory in its original form; primarily tests factual recall. | ☐ Selected |
| **Understand** | Questions requiring the student to interpret or explain a core concept, demonstrate comprehension through paraphrase, comparison, or illustration. | ☐ Selected |
| **Apply** | Questions requiring the student to use a learned concept or procedure in a new, concrete clinical or scientific situation. | ☐ Selected |
| **Analyze** | Questions requiring the student to break down information into constituent parts, identify relationships among elements, or differentiate relevant from irrelevant material. | ☐ Selected |
| **Evaluate** | Questions requiring the student to make critical judgments based on evidence, justify a clinical decision, or defend a course of action using established criteria. | ☐ Selected |
| **Create** | Questions requiring the student to synthesise existing knowledge to produce a novel solution, plan, or hypothesis not previously encountered in the same form. | ☐ Selected |

# 2. Alignment with Learning Outcome

Rate the degree to which the MCQ directly and accurately measures the intended learning outcome to which it has been assigned. Select one score on the 5-point Likert scale.

| **Score** | **Label** | **Descriptor** | **Rating** |
| --- | --- | --- | --- |
| **1** | **Inadequate** | The question measures knowledge or skills that are entirely unrelated to the target learning outcome, or is wholly misaligned with the declared curricular objective. | ☐ |
| **2** | **Poor** | The question captures only a very narrow or superficial aspect of the learning outcome; the alignment is marginal and insufficient to constitute a valid measure of the objective. | ☐ |
| **3** | **Moderate** | The question measures the learning outcome at an acceptable level; however, the alignment could be improved — the question may be too general, tangential, or partially off-target. | ☐ |
| **4** | **Good** | The question measures the learning outcome successfully and to a substantial degree; the alignment is clear and the item reflects the core intent of the objective. | ☐ |
| **5** | **Excellent** | The question fully and accurately operationalises the target learning outcome; the alignment is complete, unambiguous, and the item represents the most valid possible measure of the objective. | ☐ |

# 3. Technical Adequacy — Item-Writing Flaw Analysis

This section is designed to screen the MCQ for structural and linguistic technical flaws as defined in the NBME item-writing guidelines. Multiple criteria may be flagged. For each criterion, indicate whether the flaw is **present (Yes)** or **absent (No)**.

| **Flaw Type** | **Definition** | **Flaw Present?** |
| --- | --- | --- |
| **Long or complex options** | The answer options are unnecessarily lengthy, syntactically complex, or difficult to read relative to what the stem requires. Long correct answers may inadvertently cue examinees. | Yes ☐ / No ☐ |
| **Unnecessarily complex stem** | The question stem contains convoluted phrasing, excessive clinical detail, or indirect language that obscures the central clinical question and increases cognitive load unrelated to the construct being measured. | Yes ☐ / No ☐ |
| **Inconsistent numerical data** | Numerical values presented in the item (e.g., laboratory values, vital signs, dosages) are internally inconsistent, physiologically implausible, or mutually contradictory. | Yes ☐ / No ☐ |
| **Use of 'none of the above'** | One of the answer options is 'None of the above.' This construction is generally discouraged because it tests test-taking skills rather than content knowledge and may deflate item validity. | Yes ☐ / No ☐ |
| **Structurally inconsistent options** | The answer options are inconsistent in grammatical structure, length, or format (e.g., mixing sentence-length responses with single-word options, or mixing numerical with descriptive options). | Yes ☐ / No ☐ |
| **Negatively worded stem** | The stem is phrased using negative language (e.g., 'Which of the following is NOT…', 'Which is LEAST likely…'). Such constructions are associated with increased construct-irrelevant difficulty. | Yes ☐ / No ☐ |
| **All-inclusive options** | One or more options contain all-inclusive or absolute-implying language (e.g., 'always', 'never', 'all patients', 'in all cases'), which experienced examinees may discount regardless of content knowledge. | Yes ☐ / No ☐ |
| **Absolute terms** | Options contain absolute qualifier words (e.g., 'always', 'never', 'must', 'only') that signal or rule out the correct answer based on test-taking strategy rather than content knowledge. | Yes ☐ / No ☐ |
| **Grammatical cues** | A grammatical feature of the stem (e.g., use of 'an' before a vowel, verb tense agreement, singular/plural concordance) inadvertently reveals the correct answer option, providing a construct-irrelevant advantage. | Yes ☐ / No ☐ |
| **Conspicuous correct answer** | The correct answer option is visually or structurally distinctive compared to the distractors (e.g., it is notably longer, more detailed, more qualified, or formatted differently), allowing identification without content knowledge. | Yes ☐ / No ☐ |
| **Word repeats** | A content-specific word or phrase present in the stem also appears exclusively in one distractor (typically the correct answer), creating a lexical cue that reveals the correct option without requiring clinical reasoning. | Yes ☐ / No ☐ |
| **Common words pointing to the the correct answer** | A key term or concept in the stem has a semantic or thematic overlap with the correct answer option that is absent from the distractors, enabling content-independent identification of the correct answer through keyword matching. | Yes ☐ / No ☐ |

**Note:** Flaw categories in Section 3 are adapted from the NBME item-writing guidelines (Case & Swanson, 2002) and were translated into Turkish for administration to both expert and LLM evaluators. All evaluators received an identical rubric containing the same definitions and response format. In cases of equivocal classification under Section 1 (Cognitive Level) or Section 2 (Alignment), the lower category or score was assigned as the conservative default.
