# Supplementary material for: Assessing multiple-choice question quality in internal medicine: a comparative analysis of three large language models against expert consensus
Source: Front Med (Lausanne). 2026 Jul 9;13:1866674. doi: 10.3389/fmed.2026.1866674 (PMC13391911; doi:10.3389/fmed.2026.1866674)
Supplement: Supplementary file 2 [file Data_Sheet_2.docx]

**Large Language Model Evaluation Prompt and Output Schema**

This document reproduces the standardized prompt provided to each large language model (Claude Sonnet 4, Gemini 2.5 Flash, and Llama 3.3 70B Instruct Turbo) for the evaluation of the examination items. The evaluation was conducted in Turkish, the language of instruction and of the examination. Each prompt comprised two components: (i) a plain-text Turkish translation of the NBME item-writing (“technical flaws”) guideline, whose flaw definitions are reproduced in Supplementary Material 1; and (ii) the instruction block, item-data input, and required output schema reproduced below. Each item was submitted in an independent API call with no retained conversation history; the same prompt was repeated three times per item, and the most frequent result was adopted as the final decision. The prompt is presented first as administered (Turkish) and then in an English translation provided for the reader; the required JSON output schema follows.

**1. Instruction block**

*As administered (Turkish):*

Sana Dahiliye Stajı Bitirme Sınavı'ndan bir soru metni, doğru cevabı, seçenekleri ve sınanan kazanımı vereceğim. Sen, tıp eğitimi soru kalitesi değerlendirmesi konusunda eğitilmiş bir değerlendirici olarak hareket edeceksin. Değerlendirmeni yalnızca sağlanan kriterler ve yukarıda verilen NBME Teknik Hatalar Kılavuzu temelinde yapacaksın. Değerlendirmeni üç boyutta yapacaksın: 1. Revize Bloom Bilişsel Düzey Sınıflaması 2. Kazanım İlişkisi (5'li Likert ölçeği) 3. Teknik Uygunluk (NBME kriterlerine göre). ÇOK ÖNEMLİ: Yanıtın ilk karakteri "{" ve son karakteri "}" olmalıdır. Hiçbir açıklama, giriş metni, sonuç cümlesi veya markdown kod bloğu (```json) KULLANMA. Sadece aşağıdaki JSON yapısını döndür.

*English translation:*

I will provide a question stem, the correct answer, the options, and the targeted learning outcome from the Internal Medicine Clerkship End-of-Rotation Examination. You will act as an evaluator trained in the assessment of medical-education item quality. You will base your evaluation solely on the criteria provided and on the NBME Technical Flaws Guideline given above. You will evaluate along three dimensions: (1) Revised Bloom's cognitive-level classification; (2) Alignment with the learning outcome (5-point Likert scale); (3) Technical adequacy (according to the NBME criteria). VERY IMPORTANT: the first character of your response must be "{" and the last character must be "}". Do NOT include any explanation, introductory text, concluding sentence, or markdown code block (```json). Return only the JSON structure below.

**2. Item-data input (per item)**

The following block was appended to the instruction above for each item. Targeted learning outcomes were entered verbatim from the National Core Education Program (UÇEP).

*Template:*

SORU METNİ: [question stem]

DOĞRU CEVAP: [correct option letter, A–E]

SINANAN KAZANIM: [targeted UÇEP learning outcome, entered verbatim]

SEÇENEKLER:

A. [option A]

B. [option B]

C. [option C]

D. [option D]

E. [option E]

*Worked example (one of the 85 items):*

SORU METNİ: Demir eksikliği anemisinde Mentzer indeksi (MCV/RBC) >13 bulunur. Bu, aşağıdaki hangi iki hastalığın ayırıcı tanısında kullanılır?

DOĞRU CEVAP: D

SINANAN KAZANIM: Tanı koyabilmeli, tedavi edebilmeli, acil durumu tanımlayarak ilk tedavisini yapabilmeli, gerektiğinde uzmana yönlendirebilmeli; korunma önlemlerini (birincil, ikincil ve üçüncül korunmadan uygun olan/olanları) uygulayabilmeli.

SEÇENEKLER:

A. Talasemi – Kronik hastalık anemisi

B. Folat eksikliği – B12 eksikliği

C. Sideroblastik anemi – Aplastik anemi

D. Talasemi – Demir eksikliği anemisi

E. Talasemi – Hemolitik anemi

**3. Likert scale provided for Dimension 2 (alignment with the learning outcome)**

Anchor points provided in the prompt are shown below; the full descriptors for all five points are given in Supplementary Material 1.

*As administered (Turkish):*

1 = Kazanımla hiç ilişkili değil veya çelişkili

3 = Orta düzeyde ilişki; kazanımın bazı temel yönlerini ölçüyor ancak eksik

5 = Mükemmel ilişki; kazanımı doğrudan, tam ve uygun düzeyde ölçüyor

*English translation:*

1 = Not related to the learning outcome at all, or contradictory

3 = Moderate alignment; measures some core aspects of the outcome but is incomplete

5 = Excellent alignment; measures the outcome directly, fully, and at an appropriate level

**4. Technical-flaw evaluation instruction**

*As administered (Turkish):*

Yukarıda verilen NBME Teknik Hatalar Kılavuzu'ndaki tanımları ve örnekleri referans alarak her bir teknik hatayı değerlendir. Bir hatanın varlığından emin değilsen "Hayır" olarak işaretle (muhafazakâr yaklaşım).

*English translation:*

Evaluate each technical flaw using the definitions and examples in the NBME Technical Flaws Guideline given above (the flaw definitions are reproduced in Supplementary Material 1). If the presence of a flaw is uncertain, mark it "No" (conservative approach).

**5. Required JSON output schema**

Field names and permissible values were specified in Turkish to match the language of administration. Models were instructed to return this structure only, with no surrounding text.

{

"Soru_No": "1",

"Dusunme_Sureci": {

"Bloom_Gerekce": "[Bu sorunun hangi Bloom düzeyinde olduğunu ve neden bu düzeyde olduğunu 2-3 cümleyle açıkla. Sorunun öğrenciden ne tür bir bilişsel işlem beklediğini belirt.]",

"Kazanim_Gerekce": "[Sorunun verilen kazanımla ilişkisini değerlendir. Kazanımın hangi yönlerini ölçtüğünü veya ölçmediğini belirt. Likert puanını neden verdiğini açıkla.]",

"Teknik_Hatalar_Gerekce": "[Tespit ettiğin teknik hataları kısaca listele ve her birini neden tespit ettiğini 1 cümleyle açıkla. Hiç hata yoksa 'Teknik hata tespit edilmedi' yaz.]"

},

"Nihai_Degerlendirme": {

"Revize_Bloom_Bilisel_Duzey_Siniflamasi": "[Hatırlama|Anlama|Uygulama|Analiz|Değerlendirme|Oluşturma]",

"Kazanim_Iliskisi_Puani": [1|2|3|4|5],

"Teknik_Hatalar": {

"Uzun_veya_Karmasik_Secenekler": "[Evet|Hayır]",

"Sayisal_Verilerin_Tutarsiz_Sunulmasi": "[Evet|Hayır]",

"Belirsiz_Terimler": "[Evet|Hayır]",

"Yukaridakilerin_Hicbiri_Secenegi": "[Evet|Hayır]",

"Birbiriyle_Uyumsuz_Secenekler": "[Evet|Hayır]",

"Anlasilmasi_Guc_Soru_Metinleri": "[Evet|Hayır]",

"Olumsuz_Yapidaki_Soru_Koku": "[Evet|Hayır]",

"Dil_Bilgisi_Ipuclari": "[Evet|Hayır]",

"Butunsel_veya_Gruplandirilmis_Secenekler": "[Evet|Hayır]",

"Mutlak_Terimler": "[Evet|Hayır]",

"Goze_Carpan_Dogru_Secenek": "[Evet|Hayır]",

"Kelime_Tekrarlari": "[Evet|Hayır]",

"Yakinsama": "[Evet|Hayır]"

}

}

}

**Notes**

Field-name glossary (English): Soru_No = item number; Dusunme_Sureci = reasoning section (Bloom_Gerekce = rationale for the Bloom level, Kazanim_Gerekce = rationale for the alignment rating, Teknik_Hatalar_Gerekce = rationale for the technical-flaw judgments); Nihai_Degerlendirme = final evaluation; Revize_Bloom_Bilisel_Duzey_Siniflamasi = Revised Bloom cognitive level (Hatırlama = Remember, Anlama = Understand, Uygulama = Apply, Analiz = Analyze, Değerlendirme = Evaluate, Oluşturma = Create); Kazanim_Iliskisi_Puani = alignment score (1–5); Teknik_Hatalar = technical flaws (Evet = Yes, Hayır = No). The thirteen flaw fields correspond one-to-one to the criteria defined in Supplementary Material 1.
