# Supplementary material for: Assessing multiple-choice question quality in internal medicine: a comparative analysis of three large language models against expert consensus
Source: Front Med (Lausanne). 2026 Jul 9;13:1866674. doi: 10.3389/fmed.2026.1866674 (PMC13391911; doi:10.3389/fmed.2026.1866674)
Supplement: Supplementary file 3 [file Data_Sheet_3.docx]

Supplementary Table S1. Inter-rater reliability among the three expert evaluators

| Evaluation domain / criterion | Agreement statistic | Value | p value | Additional agreement index |
| --- | --- | --- | --- | --- |
| Bloom’s taxonomy classification | Fleiss’ κ | 0.117 | 0.001 | Unanimous: 11/85; majority: 57/85; no majority: 17/85 |
| Learning-outcome alignment | ICC(2,1) | 0.089 | 0.012 | 95% CI: -0.010 to 0.210 |
| Learning-outcome alignment | ICC(2,k) | 0.226 | 0.012 | 95% CI: -0.031 to 0.440 |
| Negatively worded stem | Fleiss’ κ | 0.751 | <0.001 | Pairwise agreement: 89.0% |
| Inconsistent numerical data | Fleiss’ κ | 0.488 | <0.001 | Pairwise agreement: 97.6% |
| Absolute terms | Fleiss’ κ | 0.283 | <0.001 | Pairwise agreement: 88.2% |
| Long or complex options | Fleiss’ κ | 0.235 | <0.001 | Pairwise agreement: 87.5% |

Supplementary Table S2. Frequency of the composite “any technical flaw” variable by evaluator

| **Evaluator** | **Items with any technical flaw, n/N (%)** | **Mean number of flaws per item** | **Median number of flaws per item** |
| --- | --- | --- | --- |
| Expert-derived consensus reference | 50/85 (58.8%) | 0.94 | 1 |
| Claude | 55/85 (64.7%) | 0.78 | 1 |
| Gemini | 56/85 (65.9%) | 0.73 | 1 |
| Llama | 7/85 (8.2%) | 0.09 | 0 |

The composite “any technical flaw” variable was coded as present if at least one of the predefined technical flaw criteria was marked as present for a given item. The expert-derived consensus reference was used as the reference standard for subsequent model comparisons.

Supplementary Table S3. Composite technical flaw analysis comparing LLMs with the expert-derived consensus reference standard

| **Model** | **TP** | **FP** | **FN** | **TN** | **Cohen’s κ** | **Agreement (%)** | **Sensitivity (%)** | **Specificity (%)** | **PPV (%)** | **NPV (%)** | **Spearman’s ρ for flaw count** | **p value** |
| --- | --- | --- | --- | --- | --- | --- | --- | --- | --- | --- | --- | --- |
| Claude | 43 | 12 | 7 | 23 | 0.528 | 77.6 | 86.0 | 65.7 | 78.2 | 76.7 | 0.547 | <0.001 |
| Gemini | 44 | 12 | 6 | 23 | 0.551 | 78.8 | 88.0 | 65.7 | 78.6 | 79.3 | 0.542 | <0.001 |
| Llama | 5 | 2 | 45 | 33 | 0.036 | 44.7 | 10.0 | 94.3 | 71.4 | 42.3 | 0.113 | 0.302 |

**Note.** TP = true positive; FP = false positive; FN = false negative; TN = true negative; PPV = positive predictive value; NPV = negative predictive value; LLM = large language model. The expert-derived consensus reference standard was used as the reference. Sensitivity indicates the proportion of expert-identified flawed items correctly identified by the LLM. Specificity indicates the proportion of expert-identified non-flawed items correctly classified as non-flawed by the LLM. Spearman’s ρ represents the correlation between the total number of technical flaws identified by each LLM and the expert-derived consensus flaw count.
